# Gait variability: methods, modeling and meaning

**DOI:** 10.1186/1743-0003-2-19

**Published:** 2005-07-20

**Authors:** Jeffrey M Hausdorff

**Affiliations:** 1 Laboratory for Gait & Neurodynamics, Movement Disorders Unit, Department of Neurology, Tel-Aviv Sourasky Medical Center, Tel-Aviv, Israel; 2 Department of Physical Therapy, Sackler School of Medicine, Tel-Aviv University, Tel-Aviv, Israel; 3 Division on Aging, Harvard Medical School, Boston, MA, USA

**Keywords:** aging, cognitive function, dual tasking, fall risk, fractals, modeling, Parkinson's disease

## Abstract

The study of gait variability, the stride-to-stride fluctuations in walking, offers a complementary way of quantifying locomotion and its changes with aging and disease as well as a means of monitoring the effects of therapeutic interventions and rehabilitation. Previous work has suggested that measures of gait variability may be more closely related to falls, a serious consequence of many gait disorders, than are measures based on the mean values of other walking parameters. The Current JNER series presents nine reports on the results of recent investigations into gait variability. One novel method for collecting unconstrained, ambulatory data is reviewed, and a primer on analysis methods is presented along with a heuristic approach to summarizing variability measures. In addition, the first studies of gait variability in animal models of neurodegenerative disease are described, as is a mathematical model of human walking that characterizes certain complex (multifractal) features of the motor control's pattern generator. Another investigation demonstrates that, whereas both healthy older controls and patients with a higher-level gait disorder walk more slowly in reduced lighting, only the latter's stride variability increases. Studies of the effects of dual tasks suggest that the regulation of the stride-to-stride fluctuations in stride width and stride time may be influenced by attention loading and may require cognitive input. Finally, a report of gait variability in over 500 subjects, probably the largest study of this kind, suggests how step width variability may relate to fall risk. Together, these studies provide new insights into the factors that regulate the stride-to-stride fluctuations in walking and pave the way for expanded research into the control of gait and the practical application of measures of gait variability in the clinical setting.

## Introduction

**Figure 1:**
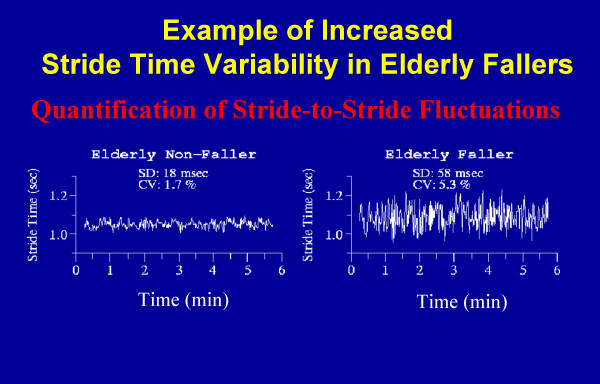
Example of the stride-to-stride fluctuations in the stride time as measured in two older adults: an older adult non-faller and an idiopathic faller. In both subjects, the stride time changes from one stride to the next. Although the mean values of the stride time are essentially identical in both subjects, the magnitude of the stride-to-stride fluctuations is much larger in the faller. SD: standard deviation; CV: coefficient of variation.

Like most physiologic signals, measures of gait are not constants but rather fluctuate with time and change from one stride to the next, even when environmental and external conditions are fixed (Figure 1). In healthy adults, these stride-to-stride fluctuations are relatively small and the coefficient of variation of many gait parameters (e.g., gait speed, stride time) is on the order of just a few percent [1-3], testimony to the accuracy and reliability of the fine-tuned systems that regulate gait. Recently, the apparently "noisy" variations in stride length, stride time and gait speed have also been shown to display a hidden and unexpected fractal-like property [4-9]. These properties of gait exhibit long-range (power-law) correlations and a "memory" effect, such that fluctuations at any given moment are statistically related to those that occur over many different time scales. When the systems regulating gait are disturbed (e.g., as a result of certain diseases), movement control may be impaired leading to increased stride-to-stride fluctuations and/or alterations in their multiscale dynamics.

The current series of the Journal of NeuroEngineering and Rehabilitation (JNER) is dedicated to gait variability. As guest editor of a collection of nine papers on this topic, I have had the opportunity to preview the wealth of information on stride-to-stride fluctuations in gait and the manifold ways in which gait variability may be analyzed. The articles in this collection cover a wide spectrum of themes ranging from methods for evaluating gait variability, animal and mathematical models investigating the factors that influence the variability of gait, and evaluations of the clinical utility of such measures. Altogether, these reports underscore the complex and fascinating nature of gait variability.

**Figure 2:**
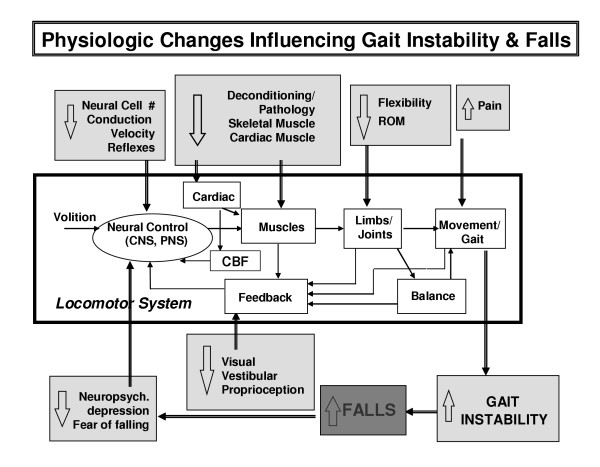
Simplified block diagram of the locomotor system. Also shown are a sample of the alterations that occur in aging and disease which affect gait stability, at least as reflected in stride time variability, and fall risk. CBF: cerebral blood flow. Modified from Hausdorff et al, *J Appl Physiol *2001.

To set the stage, it is helpful to briefly highlight previous work in this area. Earlier studies have demonstrated that:

• Gait variability is a quantifiable feature of walking that is altered (both in terms of magnitude and dynamics) in clinically relevant syndromes, such as falling, frailty, and neuro-degenerative disease (e.g., Parkinson's and Alzheimer's disease [10-19].

• The magnitude of the stride-to-stride fluctuations in stride length and step timing are unaltered in healthy older adults, whereas the dynamics of gait change with healthy aging (e.g., alterations in the fractal pattern) [1,20,21].

• Physiologic factors that affect gait dynamics include neural control, muscle function and postural control; however, more subtle alterations in underlying physiology including cardiovascular changes and mental health may also influence the variability of gait (Figure 2) [10-12,16,19,22-24].

• Improvements in muscle function and therapeutic interventions are associated with enhanced gait stability, but not always with more conventional measures of average gait velocity or cadence [12,16,25].

• Gait instability measures apparently predict falls in idiopathic elderly fallers and other populations who share an increased fall risk [2,16,17,19,26-30].

Thus, gait variability may serve as a sensitive and clinically relevant parameter in the evaluation of mobility, fall risk and the response to therapeutic interventions.

## Gait variability: a marker of fall risk

Studies of gait variability have been motivated by a number of factors. One intriguing aspect of gait variability is its relationship to fall risk. In one of the first quantitative studies of gait variability, Guimaraes and Isaacs [31] suggested that elderly fallers walked with increased gait variability, both in terms of step length and step time, compared to non-falling older adults. Indeed, one of the "holy grails" of geriatric and rehabilitation research is the identification of markers that can be used to prospectively identify older adults at greatest risk of falling. A number of studies have demonstrated that measures of gait variability may be help achieve this end [26,27,29]. Indeed, survival analyses have also shown that subjects are significantly more likely to fall sooner if gait instability measures are relatively increased at baseline, further underscoring the potential utility of such measures.

The nature of the relationships among the average gait speed, the average stride length, and the variability of these measures are critical to the study of fall risk. Although a reduced gait speed has often been viewed as a sign of fall risk, Maki showed that, at least among certain older adults, average gait speed and related measures are related to fear of falling, but not to the risk of falling per se, while measures of variability predict future falls [27]. A number of other investigations demonstrated that the degree of variability may be more closely related to fall risk than average gait speed, average stride length, and average stride time [2,26-29]. These results suggest that measures of gait variability may sometimes be more sensitive than other measures of gait, and that these measures may provide a clinical index of gait instability and fall risk. If one views gait variability as a reflection of the inconsistency in the central neuromuscular control system's ability to regulate gait and maintain a steady walking pattern, then it makes sense that measures of gait variability would be associated with instability and fall risk. A more variable gait in which the center of pressure moves over and beyond the base-of-support in a relatively uncontrolled, unstable fashion may predispose to unsteadiness and falls.

Similarly, it is important to stress that just as the assessment of the magnitude of gait variability may provide important, independent information above and beyond average values, so, too, may the investigation of the dynamics of gait variability offer additional insights. A number of studies have demonstrated this concept. Here, we briefly describe one example in which going beyond the first (the mean) and second (the standard deviation) moments proves relevant to the understanding of a disorder.

The cause of impaired gait among many older adults defies identification, even after thorough examination. This has been termed a "higher-level gait disorder" (HLGD) or "cautious gait" [32,33]. A study of the gait dynamics of these patients found that they had significantly larger (p < .0001) gait variability (the 2nd moment) compared to controls [19] and that about 50% of them reported falling. A fractal scaling index of gait was useful in discriminating fallers from non-fallers in this patient group, while all other measures (of muscle function, balance, and gait, including gait speed and stride time variability) did not [19]. These findings illustrate how going beyond conventional statistical summaries may improve discriminatory power and provide a more complete characterization of gait changes.

In the present JNER series, Brach and colleagues study the 2^nd ^moment to quantify the magnitude of stride-to-stride fluctuations and examine the relationship between gait variability and fall history in a population-based sample of more than 500 older adults. In what is probably the largest quantitative study on this question to date, too much or too little step width variability was associated with a fall history in a relatively healthy cohort of older adults who do not walk slowly (i.e., gait speed ≥1.0 m/sec). These findings raise a number of interesting questions about the relationship between variability and fall risk, and encourage the study of specific aspects of variability and their inter-relationships (e.g., step length vs. step width).

## Gait variability and heart rate variability

The strides in knowledge gleaned from studies of other physiologic systems, particularly those on heart rate variability, have also provided valuable incentive to similarly investigate gait variability [4,34-43] (see also ). The healthy heartbeat was originally thought to be quite regular and periodic, essentially the product of a single, metronomic pacemaker. Thus, for a long time, mean heart rate was regarded as the primary outcome, and fluctuations about the mean were largely ignored. It emerged from later studies, however, that the heart rate normally fluctuates, over many time scales, in a complex manner from one beat to the next [37,44]. In fact, the cardiovascular system shows erratic beat-to-beat fluctuations resembling those found in dynamical systems that are being driven away from a single equilibrium state, even under entirely healthy, resting conditions. A large body of investigations have demonstrated that there is important information hidden in the dynamics of the heart rate that can be detected using methods that examine the variability, scaling and multi-scaling properties of the heartbeat [4,39,45]. Moreover, numerous investigations have demonstrated the clinical utility of heart rate variability measures with important diagnostic and prognostic utility including the prediction of life threatening arrhythmias and mortality [46-53].

While there are obvious fundamental differences between the regulation of heart rate and the regulation of gait, the success of research into the former has spurred dynamical investigations of the latter. In the past, the fluctuations in gait were largely ignored or erroneously viewed simply as "noise". Many of the tools for quantifying heart rate variability were applied to study the stride-to-stride fluctuations in gait [5,6,8,13,19,35,36,54-56]. Of course, while both signals do share many of the same characteristics, there are several important differences: for example, increased stride time variability (i.e., the magnitude of the fluctuations) is usually a sign of pathology, while increased heart rate variability is a healthy sign. On the other hand, many of the dynamic properties of both signals are similar: heart rate and gait timing exhibit complex fluctuations reminiscent of fractals, and this property is typically altered with aging and certain diseases [4,9,19,20,47,48,54,57-59]. Challenging reports to the contrary [60], in the current series, the findings of West and Latka suggest that gait fluctuations, like the healthy heart rate, are also multi-fractal.

The parallel between gait fluctuations and heart rate variability should be considered with some caution. It would be remiss to investigate heart rate variability and not examine the average heart rate. Similarly, it would be deficient to study gait variability and disregard mean values of stride time, stride length and gait speed. These measures offer an excellent, initial description of a person's mobility and gait [61]. The lesson from the study of heart rate is that additional information can be uncovered by examining the fluctuations around the means, both in terms of the magnitude and the dynamics. The experience with heart rate also poses a challenge: pharmacologic and intervention studies have clearly identified key components that underlie the fluctuations in heart rate (e.g., the interplay between the parasympathetic and sympathetic systems). Equally fundamental studies are needed to more completely understand the physiology and patho-physiology that underlie gait variability and its dynamics.

## Methods: data acquisition and signal processing

Data acquisition and signal processing are two key areas that enable the study of gait variability. Traditional camera-based, motion analysis limits the study to a few strides and is not optimal for measuring the stride-to-stride fluctuations. A number of methods have been used to study gait under ambulatory conditions, including accelerometers, gyroscopes, foot switches, body-worn sensors and wearable computers, gait mats, and force-plate mounted treadmills or optical measurement of treadmill walking [27-29,54,62-70]. In the present series of JNER papers, Terrier and Schutz review the use of global position satellite monitoring for measuring gait. Although its time may not yet have come for routine use, this method has some important benefits, such as allowing for the determination of both the spatial and temporal measures of gait on a stride-to-stride basis.

Once the signal is acquired, questions about signal processing inevitably follow. Chau and colleagues describe challenges that arise when analyzing gait variability and present an interesting strategy for dealing with them. Their excellent review introduces the reader to different sources of variability and provides a heuristic method for summarizing various types of variability measures.

## Modeling of gait variability

A number of approaches may be applied to make sense of the various measures of gait variability. In this JNER series, Amende and colleagues report on the dynamics of gait in mouse models of Parkinson's disease, Huntington's disease and amyotrophic lateral sclerosis. In this first-ever study of the stride-to-stride fluctuations of gait in animal models of neurodegenerative processes, they demonstrate that gait changes parallel those seen in clinical studies of humans (check out the gait of these animals in on-line video). This finding supports the validity of these models and sets the stage for a novel means of studying gait dynamics. While there are of course critical differences between two and four legged locomotion, these animal models enable manipulation and invasive intervention that are not feasible in human studies, thus offering a way to identify the mechanisms that underlie changes in the stride-to-stride regulation of gait.

West and Latka take a different, complementary approach toward understanding the fluctuations in gait. Using mathematical methods, they build upon earlier nonlinear dynamics models of the fluctuations in the stride time [56,71,72] and demonstrate that these fluctuations in healthy subjects can be described using a fractional Langevin equation. It remains to be seen whether this model can be applied to data collected in animal models and how disease and aging alter model parameters.

## Gait variability, cognitive function, meaning and more

Another approach taken to gain insight into the factors that influence gait variability is to manipulate the locomotor system or specific components of the system by means of clinical studies. A priori, one might argue that stride-to-stride variability is regulated by automated processes and requires minimal cognitive resources. This argument is consistent with the report of Maki [27], demonstrating that variability was related to fall risk, but not to fear of falling. Indeed, studies of dual tasking found that gait speed slowed when healthy subjects, young and old, performed a secondary dual task during walking, while the variability of stride and swing timing was unchanged, even when subjects simultaneously walked and subtracted 7's serially, a challenging cognitive task [73,74]. In contrast, dual tasking not only reduced gait speed, it also increased variability among patients with impaired automaticity (e.g., Parkinson's disease patients) [17,73-75]. These findings are in line with the view that the regulation of variability is normally automated and requires minimal cognitive input. However, when automaticity is impaired (e.g., in the presence of pathology, cognitive tasks affect gait variability. One recent investigation disputed the concept of automatic regulation and suggested that stride time variability is related to specific cognitive processes, namely executive function [76]. In the present series, papers by Beauchet and colleagues and Grabiner and Troy describe the effects of a secondary, dual task on the gait variability in healthy young adults. One study suggests that there is no effect on stride length variability, while there is a small increase in stride time variability due to changes in mean gait speed. The second paper suggests that stride width variability becomes *reduced *during dual tasking. These interesting findings raise the question: "why?" and call for a more all-embracing understanding of the mechanisms that control gait variability and a "smooth" gait.

When dealing with this question, the complex relationships between gait speed and measures of variability of gait should be considered. When all other variables are kept constant, studies in young adults have demonstrated a U-shaped relationship between stride length (speed and/or cadence) and measures of gait variability. Minimal variability occurred near the usual walking speed and cadence [77-79], where energy costs of walking are also minimal and head stability is maximal [80,81]. Thus, when investigating gait and the factors that influence variability, it is important to take into account the possibility that any observed group differences or responses to intervention are simply a result of changes in gait speed. In many cases, however, it is possible to demonstrate that variability parameters are regulated independently of mean values (e.g., of stride length and stride time) [78]. For example, in the present series, Kessler and colleagues show that healthy controls and patients with a HLGD reduce their stride length and walk more slowly when they are asked to walk in conditions of minimal lighting. While variability measures *increased *among the patients, control subjects evidenced *no change*, even though they did walk more slowly in near darkness.

A potential way of separating values of variability from those of mean stride length and speed is described by Frenkel-Toledo and colleagues in the present series. They show that swing time variability is larger in patients with Parkinson's disease compared to healthy controls and that swing time variability is insensitive to changes in gait speed in both groups. Perhaps this measure can be used as a speed-independent measure of variability to help to unravel the mechanisms that influence the stride-to-stride fluctuations of gait and to identify measures with clinical utility that are not influenced by gait speed. Interestingly, a recent report observed a dissociation between left and right swing time variability in patients with Parkinson's disease who have a severe gait disturbance, i.e., freezing of gait [82], further demonstrating the potential utility of measures of swing time variability.

## Outstanding issues

The investigations reported in this special series on gait variability advance our understanding of an intriguing aspect of gait: the ability of the healthy neural control system to fine tune the stride-to-stride fluctuations of walking to a remarkable degree. At the same time, they delineate a number of important questions that remain to be resolved by future studies. For example, several reports highlight the differences between measures of the mean, the variance and the dynamics. A theoretical framework is needed to understand these differences. One possible explanation for the difference between the results of the study by Brach and colleagues and those of previous studies relates to the question: how much is enough? In order to obtain reliable and meaningful measures of variability, how many strides need to be studied? Owings and Grabiner [64] suggest that hundreds of strides are required to accurately estimate step kinematic variability for treadmill walking. The number needed for walking on level ground is undetermined. If variability measures are to be used in the clinic, more research is required to determine the trade-off between accuracy, reliability, validity and clinical utility.

The question of how many strides to measure highlights the need for the development of standards and reference values. Standards were set to define minimum data acquisition requirements (e.g., sampling rate) to promote research and the clinical implementation of heart rate variability measures [83]. While there may be some debate about the exact values, the defining of standards greatly enhances the quality of the data and the ability to interpret and compare studies that use a given tool. Similarly, well-established reference values and norms are needed in different age groups and populations in order to promote interpretation and clinical references. Heart rate databases in different populations, complete with annotations and medical information, are widely available (e.g., see ), significantly advancing the sharing of methods and interpretation. Similar open-access database efforts would greatly help the study of gait variability and the development of clinical measures, but this must await the establishment of minimum standards for data collection and validation of the means of comparing data from different measurement systems.

The studies by West and Latka and Brach and colleagues also return us to hypotheses originally put forth by Gabell and Nayak over two decades ago [1]. They speculated that stride time variability reflects gait timing mechanisms and the pattern generator of gait, while variability of support time and step width more closely reflect balance control. Future studies are needed to unravel the various aspects of gait variability and their nonlinear interactions (in this respect, the potential of the animal models comes to fore), to identify the mechanisms that are responsible for each of the complementary measures of the stride-to-stride fluctuations in gait, and to work out the relationship between balance control and gait variability. The basal ganglia and dopamine-sensitive networks apparently participate in the regulation of gait variability while visual feedback apparently does not play a critical role in healthy adults. We lack, however, a good understanding of the neural center(s) that generates and regulates gait timing and are left to speculate why the maintenance of gait variability may be influenced by cognitive challenges, at least certain types under specific conditions. It has become clear that more sinus rhythm heart rate variability is (generally) "good", while more stride time variability is (generally) "bad" The final words on the value and interpretation of the variability of multiple other aspects of gait (e.g. step width variability), their inter-dependence and the relationship to the variability of other motor control tasks await the results of future studies [63,65,68,84-88].

## Conflict of interest Statement

The author(s) declare that they have no competing interests.
